# Three new mycoviruses identified in the apple replant disease (ARD)-associated fungus *Rugonectria rugulosa*

**DOI:** 10.1007/s11262-022-01924-6

**Published:** 2022-07-16

**Authors:** Tom P. Pielhop, Carolin Popp, Dennis Knierim, Paolo Margaria, Edgar Maiß

**Affiliations:** 1 Institute of Horticultural Production Systems, Department of Phytomedicine, Leibniz University Hannover, Herrenhäuser Str. 2, 30419 Hannover, Germany; 2 Leibniz Institute DSMZ, German Collection of Microorganisms and Cell Cultures, Inhoffenstraße 7 B, 38124 Brunswick, Germany

**Keywords:** Mycovirus, Quadrivirus, Mitovirus, Unclassified dsRNA virus, *Rugonectria rugulosa*, Apple replant disease

## Abstract

In this study, three new mycoviruses were identified co-infecting the apple replant disease (ARD)-associated root endophyte *Rugonectria* *rugulosa*. After dsRNA extraction, six viral fragments were visualized. Four fragments belong to a quadrivirus, which has a genome size of 17,166 bp. Each of the fragments of this quadrivirus has a single ORF encoding a protein. Two of these proteins are coat protein subunits, one ORF encodes the RdRp, and one protein has an unknown function. This virus was tentatively named rugonectria rugulosa quadrivirus 1 (RrQV1) as a member of the proposed new species *Quadrivirus rugonectria*. Another fragment represents the dsRNA intermediate form of a + ssRNA mitovirus with a genome size of 2410 nt. This virus encodes an RdRp and is tentatively called rugonectria rugulosa mitovirus 1 (RrMV1). RrMV1 is suggested as a member of a new species with the proposed name *Mitovirus rugonectria*. The sixth fragment belongs to the genome of an unclassified dsRNA virus tentatively called rugonectria rugulosa dsRNA virus 1 (RrV1). The monopartite dsRNA genome of RrV1 has a length of 8964 bp and contains two ORFs encoding a structure/gag protein and an RdRp. Full genomic sequences were determined and the genome structure as well as molecular properties are presented. After phylogenetic studies and sequence identity analyses, all three isolates are proposed as new mycoviruses. The results help to improve the understanding of the complexity of the factors involved in ARD and support the interest in mycoviral research. Subsequent analyses need to focus on the impact of mycoviruses on the biology and pathogenicity of ARD-associated fungi. The results of such studies could contribute to the development of mitigation strategies against the disease.

**Supplementary Information:**

The online version contains supplementary material available at 10.1007/s11262-022-01924-6.

## Introduction

Since the first proven infection of a fungus with a mycovirus in 1962, these viruses and their effect on the host have increasingly been part of intensive research [1]. To date, viral infections have been reported in all main fungal taxa [2, 3]. The majority of these identified viruses have RNA genomes, mostly appearing to be constituted of dsRNA [4]. DsRNA mycoviruses are assigned to 8 taxonomic families and one genus, whereas those with an ssRNA genome are separated in 12 families, of which 11 families are based on + ssRNA viruses and just one on -ssRNA viruses [5]. Up to now, less is known about DNA mycoviruses. Since the first report of a geminivirus-related DNA mycovirus in 2010, just a few more were detected [6–8]. Mycoviral infections are usually persistent, but often seem to not affect the phenotypes of the respective host [5]. However, many mycoviruses are known to have either a hypo- or hypervirulent effect on their host fungi [9–15]. In the case of hypovirulence, an infection causes a decrease in host pathogenicity. On the contrary case of hypervirulent viral infections, the pathogenic effect of the host fungus is enhanced. In this study, viral infections of an endophytic *Rugonectria* *rugulosa* isolate were investigated. The fungus was isolated from apple plant roots (*Malus* *x domestica*, Borkh.; M26), suffering from apple replant disease (ARD). ARD is a worldwide problem, occurring in orchards when apple is planted repeatedly. The disease is caused by plant reactions due to changes in their (micro-) biome [16]. When affected, apple orchards can lose about 50% profitability by reduction of yield, tree vigor and a possible delay of 2–3 years, after which the trees begin to bear fruit [17]. The causes for ARD are highly complex and include oomycetes, bacteria, nematodes, fungi, and others [16].

In the search for factors of ARD etiology, Nectriaceae species such as *R. rugulosa* are found repetitively. By sampling apple roots grown in ARD suffering soils, *R. rugulosa* was found together with related Nectriaceae species in all soils of three ARD affected sites in Germany [18]. Thus, bioassays have shown that fungi of this family can cause ARD symptoms after isolation and re-inoculation [19].

Since ARD is still not fully understood, all contributing factors, such as mycoviruses, have to be taken into consideration. Understanding a possible hypo- or hypervirulence of the occurring mycoviruses might help to investigate on potential applications in biological control and disease management. Thus, the research on mycoviruses is an important component in the development of strategies to mitigate complex plant diseases such as ARD. However, many phytopathogenic fungi are involved in the etiology of the disease and it can be assumed that many of those fungi also carry unidentified viruses. Therefore, the main objective of this study is to give a first insight into the fungal virome of one of the most recurring fungi involved in the etiology of ARD.

Because of the dominance of dsRNA mycoviruses, dsRNA was extracted from *R. rugulosa* and sequenced by an Illumina system. Sequences were de novo assembled and completed by RACE (Rapid amplification of cDNA ends), followed by annotation of the molecular features of the individual viruses and phylogenetic analyses. The data and full genomic sequences of the three newly identified mycoviruses, presented in this study, are essential for a deeper understanding of the virus–host relationship and form the basis for further research projects.

## Materials and methods

### Fungal material

The *R. rugulosa* isolate, used in this study was isolated from ARD suffering, in vitro propagated M26 roots (*Malus x* *domestica*, Borkh.), which were grown for 8 weeks in soil from the ARD affected site Ellerhoop (Chamber of Agriculture Schleswig–Holstein, Germany, 53°42′51.7″N, 9°46′12.5″E) [20]. Before isolation, fine root surfaces were disinfected (70% Ethanol 30 s, followed by 7.5 min 2% NaOCl). Root pieces of 1 cm length were plated on water agar (50 µg mL^−1^ penicillin, 10 µg mL^−1^ rifampicin, 25 µg mL^−1^ pimaricin). The outgrown endophytic fungi were separated and cultivated on 2% malt extract agar (MEA). For nucleic acid extraction, the fungi were propagated in 2% malt extract broth for 2 weeks and mycelium was ground in liquid nitrogen. *R.* *rugulosa* was identified by PCR and Sanger sequencing, as described by Crous et al. [21], using primers for the histone H3 gene (CYLH3F: 5ʹ AGGTCCACTGGTGGCAAG 3ʹ; CYLH3R: 5ʹ AGCTGGATGTCCTTGGACTG 3ʹ) [22]. The sequencing was performed at Microsynth Seqlab (Göttingen, Germany). The resulting sequences were analyzed by NCBI BLASTn.

The *R. rugulosa* isolate investigated in this study was named No4. To test the infectivity of No4 after isolation, M26 plants were re-infected using a soil-free inoculation assay as described by Popp et al., 2019 [19]. The plants showed reduced growth, as well as typical blackening in microscopic analyses of fine roots after 5 weeks.

### Extraction of nucleic acids

DsRNA for Illumina sequencing was extracted from 20 g ground fungal material, stored at − 80 °C, based on a modified protocol of Morris and Dodds [23] as described by Lesker et al. [24], apart from using a different cellulose (acid-washed powder for column chromatography [Merck; Darmstadt, Germany; product nr. 22,184]). 20 mL eluate was digested first with 20 U Rnase T1 (Roche; Basel, Switzerland) and then with 40 U DNAse I (Roche; Basel, Switzerland) at 37 °C for 30 min each. DsRNA extracts were centrifuged and suspended in 25 µL Tris (5 mM). Subsequently, 20 µL extract was checked with 5 µL of GelRed® (Biotium; Fremont, CA, USA) dye in 1.5% agarose gel electrophoreses. For virus detection by RT-PCR and RNA end determination, a simpler protocol for whole nucleic acid extraction was used, following the protocol of Menzel et al. [25].

### Illumina sequencing

A Nextera XT Library Preparation Kit was used to prepare an Illumina library from double-stranded cDNA, obtained by cDNA synthesis of the dsRNA extract and second-strand synthesis with random octamer primers. The library was sequenced at the Leibniz-Institute DSMZ on a NextSeq instrument as paired-end reads (2 × 151 bp). The raw reads were trimmed and de novo assembled with Geneious v. R11.1 software (Biomatters; Auckland, New Zealand) using an in-house established workflow, followed by local BLASTn and BLASTp alignments of the assembled contigs against a custom database of NCBI nuclear-core reference sequences. The identified mycovirus contigs were ordered and trimmed according to reference sequences to determine the nearly complete genome sequences. The sequence information was used to design primers for virus detection by RT-PCR and determination of the extreme terminal ends of the genomes by RACE.

### Virus detection with RT-PCR

RT-PCR protocols were adapted for the detection of each of the genomic viral segments. For cDNA synthesis, 4 µL dsRNA extract, 1 µL cDNA primer [10 µM (Table 1); salt-free; Eurofins Genomics; Ebersberg, Germany] and 5 µL A.bidest were mixed and heated up to 95 °C for three minutes to separate the dsRNA strands. 50 U Maxima H Minus Reverse Transcriptase (Thermo Fisher Scientific™; Waltham, MA, USA), 20 U RiboLock RNase Inhibitor (Thermo Fisher Scientific™; Waltham, MA, USA), 1 µL dNTPs (10 mM each; Thermo Fisher Scientific™; Waltham, MA, USA) and 4 µL 5X RT-buffer (250 mM Tris–HCl (pH 8.3), 250 mM KCl, 20 mM MgCl2, 50 mM DTT; Thermo Fisher Scientific™; Waltham, MA, USA) were added subsequently and adjusted to 20 µL with A.bidest. The cDNA synthesis started with 60 min at 50 °C, followed by 15 min at 55 °C, 15 min at 60 °C, and 5 min at 85 °C. PCR was performed with 5 µL 2 × Phusion Flash High-Fidelity PCR Master Mix (Thermo Fisher Scientific™; Waltham, MA, USA), 1 µL of each specific primer [forward and reverse, 10 µM each, salt-free; Eurofins Genomics; Ebersberg, Germany (Table 1)], 2 µL cDNA and 1 µL A.bidest. PCR was performed with an initial denaturation of 15 s at 98 °C, followed by 34 cycles (denaturation: 98 °C, 5 s; annealing: primer T_A_, 5 s; elongation: 72 °C, 15 s / 1000 bp amplicon), and a final elongation of 300 s at 72 °C. Amplicons were visualized by 1.0% agarose electrophoresis and sent to Microsynth Seqlab (Göttingen, Germany) for Sanger sequencing [22].

**Table 1:** Primers used for RT-PCR detection with expected amplicon sizes of rugonectria rugulosa quadrivirus 1 (RrQV1), rugonectria rugulosa mitovirus 1 (RrMV1) and rugonectria rugulosa dsRNA virus 1 (RrV1)

Viral segment	Type	Name	Sequence (5ʹ– > 3ʹ)	Amplicon size (bp)
RrQV1 RNA1	cDNA	Quadri1_DetcDNA	GCTTCAACCTCATCTGCC	990
	PCR forward	Quadri1_Dets	CGCACCTGCAACTCTATAC	
	PCR reverse	Quadri1_Detas	TCTCTCCCATAACTTCCACTC	
RrQV1 RNA2	cDNA	Quadri2_DetcDNA	CATCAACGTAGTCACAGGAAG	1019
	PCR forward	Quadri2_Dets	ACATACACACAACCAAACACC	
	PCR reverse	Quadri2_Detas	CCAACTACCTGCCAGACAC	
RrQV1 RNA3	cDNA	Quadri3_DetcDNA	CTTCGGCAAGGCTAGAAAC	1396
	PCR forward	Quadri3_Dets	GCACATACACAACAGCACC	
	PCR reverse	Quadri3_Detas	CGCCTTCCACAAAACACTTC	
RrQV1 RNA4	cDNA	Quadri4_DetcDNA	CTGACCTAACGACTTTGATCC	1089
	PCR forward	Quadri4_Dets	GCAACCTGTTACGCTTACC	
	PCR reverse	Quadri4_Detas	CGCTTCCATCTCTGTCTTTC	
RrMV1	cDNA	Mito_Detc	TGATTGAATCACGGTCCTTTC	1054
	PCR forward	Mito_Dets	TCACCAAACAATGAGAAGCC	
	PCR reverse	Mito_Detas	AAAGCGACAGCAGTTATACC	
RrV1	cDNA	Rugo_Detc	AAGAGGGGATAAGGTGACCG	938
	PCR forward	Rugo_Dets	ATCTTCTTACACCCCACCC	
	PCR reverse	Rugo_Detas	TGCCGCCCTATACTATGAC	

### RNA end determination

The ends of dsRNAs were determined by RACE with an adapted protocol, based on the method described by Frohman et al. [26]. 3ʹ-ends of both, dsRNA sense and antisense strands were analyzed. Reverse transcription followed the described protocol for virus detection, with different cDNA primers (10 µM each, salt-free; Eurofins Genomics; Ebersberg, Germany; Table 2). For tailing 3 µL cDNA was mixed with 20 U Terminal Deoxynucleotidyl Transferase (TdT; Thermo Fisher Scientific™; Waltham, MA, USA), 4 µL 5 × TdT Reaction Buffer (500 mM potassium cacodylate (pH 7.2), 10 mM CoCl2, 1 mM DTT; Thermo Fisher Scientific™; Waltham, MA, USA), 1 µL of either dATP, dCTP, dGTP, or dTTP (100 mM, Thermo Fisher Scientific™; Waltham, MA, USA) and 11 µL A.bidest. The mixture was incubated for 30 min at 37 °C followed by 10 min at 70 °C. For each RNA end, at least two different tails were used in different reactions. The subsequent PCR was performed as the one for virus detection with a different primer set (poly-n primer; nested primer; 10 µM each, salt-free; Eurofins Genomics; Ebersberg, Germany; Table 2).

**Table 2:** Primers for 3ʹ- and 5ʹ-end determination by RACE of rugonectria rugulosa quadrivirus 1 (RrQV1), rugonectria rugulosa mitovirus 1 (RrMV1) and rugonectria rugulosa dsRNA virus 1 (RrV1)

Viral segment	Type	Name	Sequence (5ʹ– > 3ʹ)
–	Poly-A	Poly-A16	CCTCGGGCAGTCCAAAAAAAAAAAAAAAA
–	Poly-G	Poly-G15	CTCAAACAGTCACGGGGGGGGGGGGGGG
–	Poly-C	Poly-C14	ATCCTGCAGGCGCGCCCCCCCCCCCCCC
–	Poly-T	Poly-T18	CCTCGGGCAGTCCTTTTTTTTTTTTTTTTTT
RrQV1 RNA1 5ʹ-end	cDNA	Quadri1_5e_cDNA	GCTACTCTTAGTCGCTAACATC
	Nested	Quadri1_5e_nested	ACGCCACTCGCAAGATAC
RrQV1 RNA1 3ʹ-end	cDNA	Quadri1_3e_cDNA	GCAAGATTCAAGAGCTACCC
	Nested	Quadri1_3e_nested	AGAACCACGCAGAGATGAAG
RrQV1 RNA2 5ʹ-end	cDNA	Quadri2_5e_cDNA	GCAGCCAGCATAGACAAAG
	Nested	Quadri2_5e_nested	CGAGAACACCCAACCAGTAG
RrQV1 RNA2 3ʹ-end	cDNA	Quadri2_3e_cDNA	CCACGAGAGAAGCATTTGAC
	Nested	Quadri2_3e_nested	CTATGCACCTGCTACACAC
RrQV1 RNA3 5ʹ-end	cDNA	Quadri3_5e_cDNA	TCCTTCCTCGACCTAATTGAC
	Nested	Quadri3_5e_nested	TCTATCTCACACAGCTCACC
RrQV1 RNA3 3ʹ-end	cDNA	Quadri3_3e_cDNA	GAAGAGTAGAAGCAGACAACC
	Nested	Quadri3_3e_nested	CACAGAAGAGTAAAGGAAGCAG
RrQV1 RNA4 5ʹ-end	cDNA	Quadri4_5e_cDNA	GCAAGCATTGCATTGTCTCC
	Nested	Quadri4_5e_nested	TCTTGACACTCAGCGGTTC
RrQV1 RNA4 3ʹ-end	cDNA	Quadri4_3e_cDNA	TGAAAACTGCACAGGCAC
	Nested	Quadri4_3e_nested	ACAGCAGACGATACACGAC
RrMV1 5ʹ-end	cDNA	Mito_5e_cDNA	CGGACTGTCTTCATGTACTTG
	Nested	Mito_5e_nested	GGAGAAATCCAAATGGAATGGC
RrMV1 3ʹ-end	cDNA	Mito_3e_cDNA	GTCAGGTATAACTGCTGTCG
	Nested	Mito_3e_nested	GACCGTGATTCAATCATAGTCC
RrV1 5ʹ-end	cDNA	Rugo_5e_cDNA	GTGGAGAAAGGAGAAAACAGG
	Nested	Rugo_5e_nested	ATAGGAGAGGTTGAGGGTGG
RrV1 3ʹ-end	cDNA	Rugo_3e_cDNA	GTATTAACTCCGCAACGACC
	Nested	Rugo_3e_nested	TTAGTGCCACCCTTCAACC

### Phylogenetic analyses

Phylogenetic analyses were performed with several mycoviruses of the families *Totiviridae*, *Quadriviridae*, *Chrysoviridae*, *Mitoviridae*, and ten unclassified dsRNA *Riboviria* viruses. Before constructing a phylogenetic tree, amino acid sequences of the RNA-dependent RNA polymerase of all viruses were aligned, using the MUSCLE algorithm in MEGA X [27, 28]. Parameters were set to default (gap opening: − 2.9, gap extension: 0). After the initial alignment, highly conserved sequences were selected, referring to the segment A(679)–E(1066) of NC_016760 [29]. The final alignment was performed with the set of chosen segments and default parameters, using the MUSCLE algorithm again. A maximum-likelihood tree was calculated, using the bootstrap method with 1000 replications and the Le_Gascuel_2008 substitution model with discrete Gamma distribution (LG + G) [30]. The number of discrete gamma categories was set to 5 and for the data subset, all sites were used. Pairwise alignments of all segments of the families *Quadriviridae*, *Mitoviridae*, and the unclassified dsRNA viruses were done to calculate sequence identities by using the EMBOSS/Needle tool [31].

### UTR alignment, secondary structures, and motifs

Conserved UTR-sequences of the proposed quadrivirus RrQV1 were analyzed by alignments of the four 5ʹ- and 3ʹ-ends and presented with the GeneDoc Software (National Resource for Biomedical Supercomputing, Pittsburgh, PA, USA). Secondary structure predictions of the proposed mitovirus RrMV1 were computed with the RNAfold WebServer (Institute for Theoretical Chemistry, University of Vienna) [32, 33]. Conserved motifs within the genomic RNAs were identified by using the NCBI Conserved Domain Search tool with default options (database: CDD v3.19–—58,235 PSSMs, expected value threshold: 0.01) [34].

## Results and discussion

### Identification of new mycoviruses

**Fig. 1:**
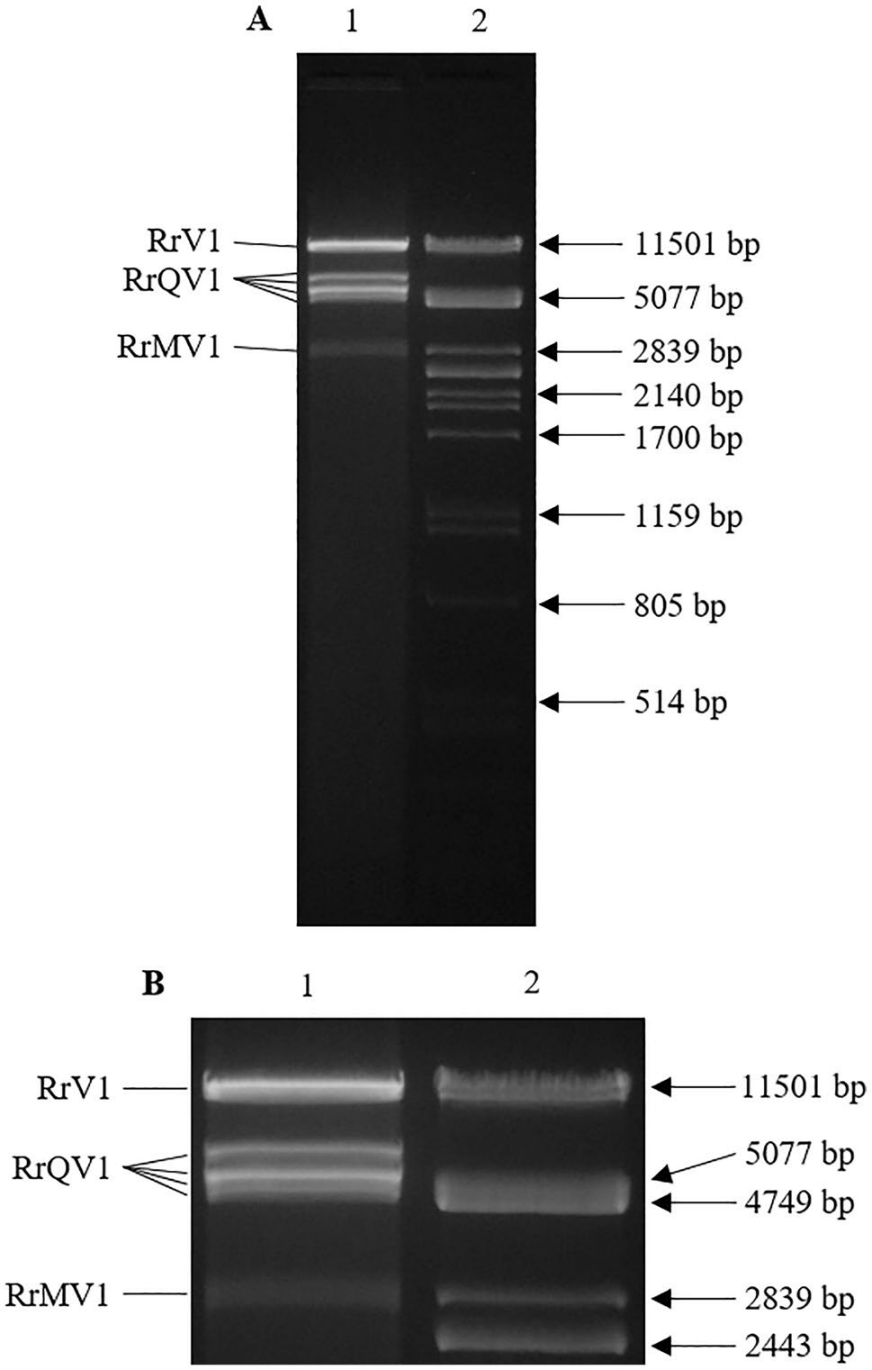
1.5% Agarose gel electrophoresis of dsRNA, isolated from *Rugonectria rugulosa*. Fragments correspond for rugonectria rugulosa quadrivirus 1 (RrQV1), rugonectria rugulosa mitovirus 1 (RrMV1), and rugonectria rugulosa dsRNA virus 1 (RrV1). Nucleic acid stained with GelRed®. Lane 1: dsRNA, Lane 2: *Pst*I digested λ phage DNA. **A** Original gel, **B** Detailed view of dsRNA fragments

After dsRNA extraction of *R. rugulosa*, agarose gel electrophoresis enabled the visualization of six fragments (Fig. 1). Illumina sequencing and de novo assembly led to six consensus sequences, which were assigned to different virus families by BLAST. Accordingly, four fragments represent the different segments of a quadrivirus. One fragment belongs to a member of the family *Mitoviridae* and another one to an unclassified dsRNA virus. Each of the six RNAs could be detected in *R. rugulosa* by RT-PCR. End determination by 5ʹ- and 3ʹ-RACE led to full genomic RNAs. The four segments of genomic RNAs of the quadrivirus—with the proposed name rugonectria rugulosa quadrivirus 1 (RrQV1)—have sizes of 4897 bp, 4312 bp, 4153 bp, and 3804 bp and each RNA encodes a single open reading frame (ORF). The mitovirus with the suggested name rugonectria rugulosa mitovirus 1 (RrMV1) consists of one RNA with a length of 2410 nt and one ORF. The unassigned virus with the proposed name rugonectria rugulosa dsRNA virus 1 (RrV1) has one genomic RNA, 8964 bp in length, which encodes two ORFs.

Subsequent comparison of genome sizes determined from sequence analyses with sizes from the agarose gel of dsRNA extractions reveals that the fragments in the gel, compared to the λ-phage DNA ladder, were all higher than expected. Such a shift can occur when using GelRed® as dye for nucleic acids in agarose gels. Although it is relatively non-toxic, it leads to artifacts in agarose gel electrophoreses. The more nucleic acids are present, the stronger these artifacts become and the more difficult a size determination of the fragments [35]. Additionally, dsRNA generally migrates more slowly in agarose gels than DNA because of a lower overall charge density of RNA, which results from condensation and the development of secondary structures [36, 37]. Condensation is comparatively more likely in RNA because the phosphate groups are more closely adjacent than in DNA [38]. Compared to the other viruses, the fragment of the mitovirus is fainter visible in the gel. Since mitoviruses are + ssRNA viruses, it must be assumed that this fragment is not the genomic but its dsRNA intermediate, which occurs in all + ssRNA viruses [39].

### Genome organization

**Fig. 2:**
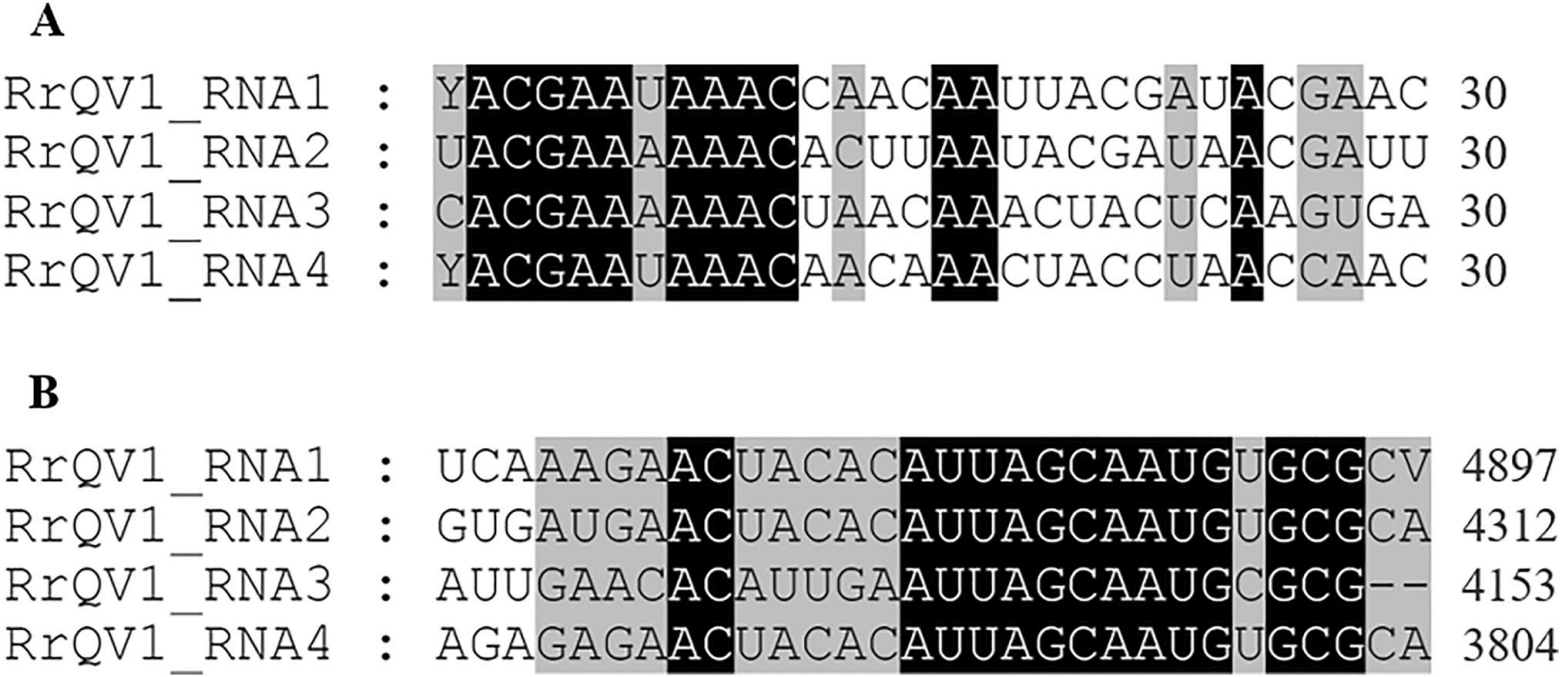
Alignment of **A** 5ʹ-UTRs and **B** 3ʹ-UTRs of RrQV1. Numbers next to the sequences represent the nucleotide position, mapped with GeneDoc 2.7. Black boxes are conserved domains. Boxes in gray have one dissimilar nucleotide in maximum

The genome of RrQV1 consists of 17,166 bp, split into four ORFs on four dsRNA segments. 5ʹ-untranslated regions (UTRs) are 49 bp to 62 bp long and 3ʹ-UTRs range from 37 to 380 bp. With sizes ranging from 3804 to 4897 bp, the genomic RNAs meet the requirements for segments of quadriviruses, which should range from 3.5 to 5 kbp [29]. Proteins encoded by the ORFs comprise 1591 amino acids (aa, p1), 1384 aa (p2), 1351 aa (p3), and 1120 aa (p4). These proteins have a calculated molecular mass of 175.5 kDa (p1), 150.9 kDa (p2), 148.9 kDa (p3), and 119.5 kDa (p4), respectively. Protein p1 has a so far unknown function, whereas p2 and p4 are coat protein (cp) subunits. P3 is the RNA-dependent RNA polymerase (RdRp) and has a conserved RT-like domain at aa651–aa1112. Upstream of the respective ORFs within the 5ʹ-UTRs of RrQV1, (CAA)_n_ repeats are localized, which may serve as translational enhancers [29, 40]. The first 11 nucleotides at the 5ʹ-end and the last 16 nucleotides at the 3ʹ-end of RrQV1 are highly conserved (5ʹ YACGAAWAAAC…AUUAGCAAUGYGCGCV 3ʹ). This holds true for all four segments, with exception of the 3ʹ-end of RNA 3, which lacks the last two nucleotides. An alignment of the first and last 30 nucleotides of the RrQV1 segments is shown in Fig. 2.

**Fig. 3:**
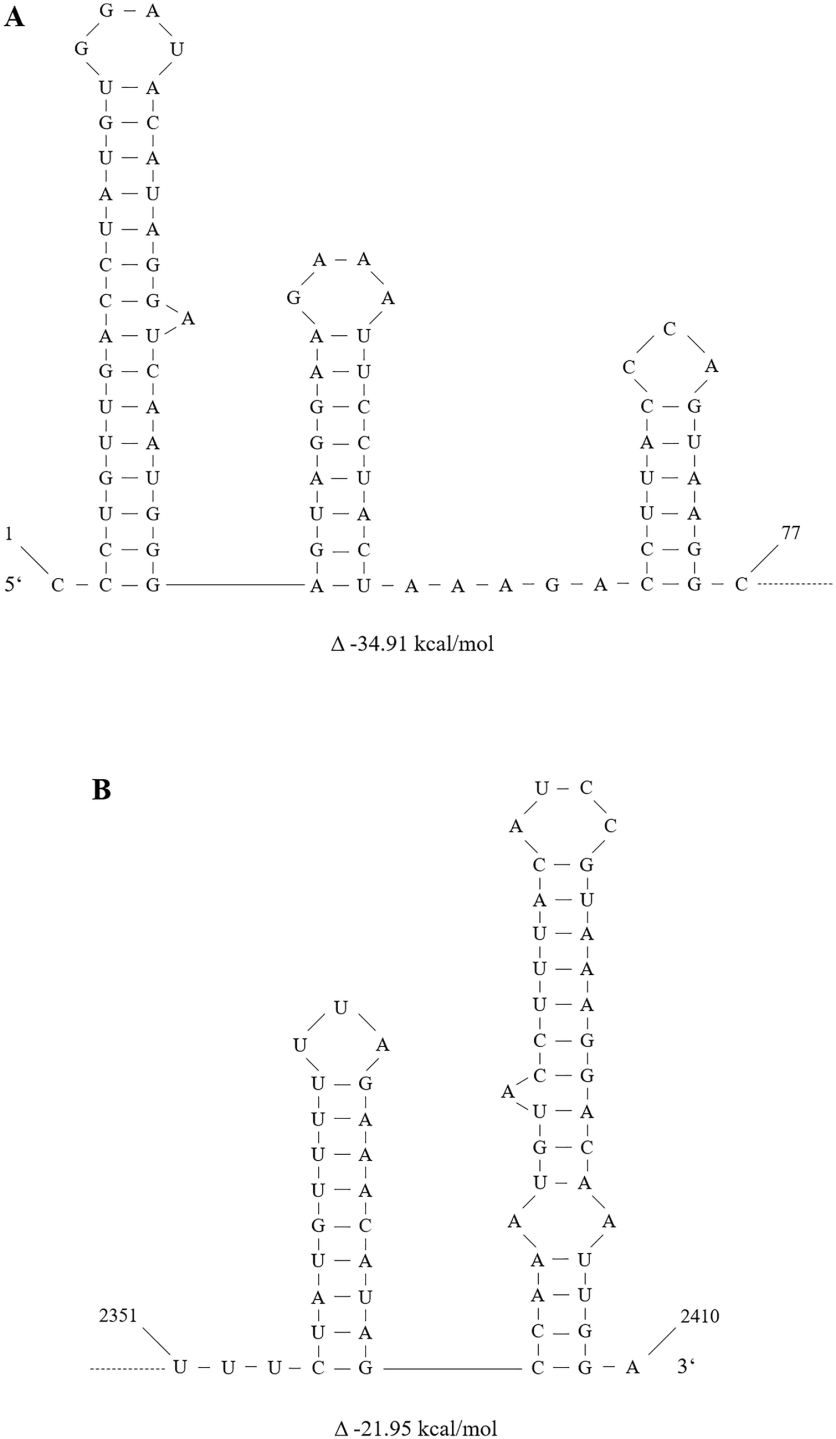
Predicted secondary structures for 5ʹ-end (**A**) and 3ʹ-end (**B**) of RrMV1*,* calculated with the RNAfold webserver [32, 33]. Change of energy level is given in Δ kcal/mol below the respective graphic

The genome of the mitovirus RrMV1 is monopartite with a 2410 nt + ssRNA. One single ORF is flanked by a 197 nt 5ʹ-UTR and a 68 nt 3ʹ-UTR. It encodes an 83.9 kDa RdRp with a length of 714 aa. A conserved mitovirus-RNA-Polymerase domain was found at position aa170-aa633. Modeling the structure of the terminal sections of the + ssRNA of RrMV1 resulted in three stem-loop structures at the 5ʹ-end with a negative energy change of − 34.91 kcal mol^−1^ and two stem-loops at the 3ʹ-end with − 29.95 kcal mol^−1^. The plain structure predictions are shown in Fig. 3. This formation of secondary structures at the ends of the genome is characteristic in mitoviruses and presumably serves to protect the naked genomic RNA from enzymatic degradation in the host cells [41, 42].

**Fig. 4:**
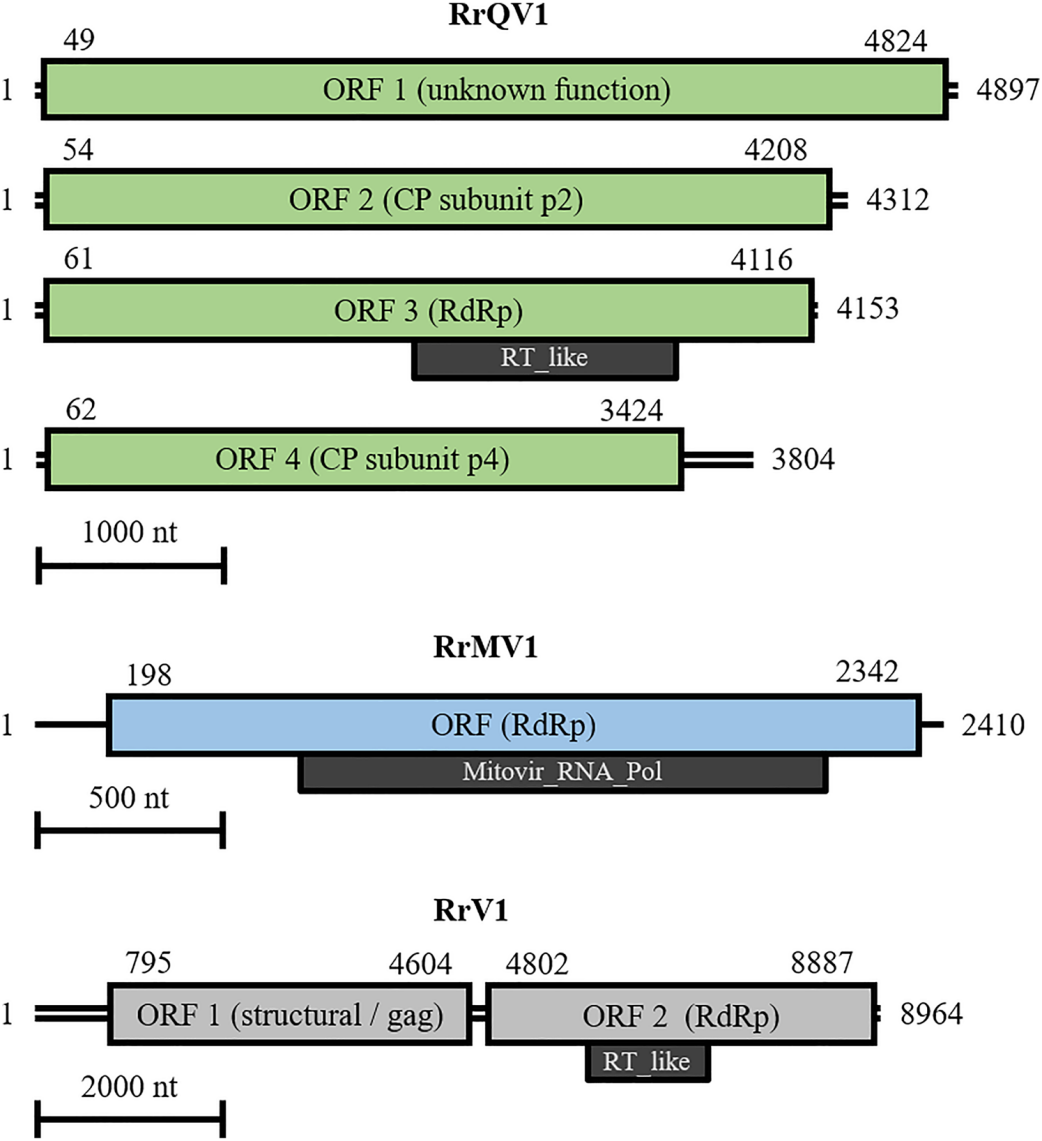
Scaled genome maps. Green: rugonectria rugulosa quadrivirus 1 (RrQV1); blue: rugonectria rugulosa mitovirus 1 (RrMV1); light gray: rugonectria rugulosa dsRNA virus 1 (RrV1). Digits indicate nucleotide positions of 5ʹ-ends, ORFs, and 3ʹ-ends. Boxes represent ORFs with their function (encoding protein) in brackets. Dark gray boxes: Conserved RdRp domains. Lines represent genomic RNAs. Single line: ssRNA, double lines: dsRNA. Scale bars indicate 1000 nt, 500 nt, and 2000 nt, related to the respective virus

The unclassified virus RrV1 has a monopartite 8964 bp dsRNA genome. A 794 bp 5ʹ-UTR and a 77 bp 3ʹ-UTR surround two ORFs with lengths of 3810 nt and 4086 nt. The translated proteins are a proposed 141.0 kDa structural/gag protein (p1) with a length of 1269 aa and a 151.1 kDa RdRp with a length of 1361 aa. A scaled genome map of the three viruses is shown in Fig. 4.

### Phylogenetic analyses

**Fig. 5:**
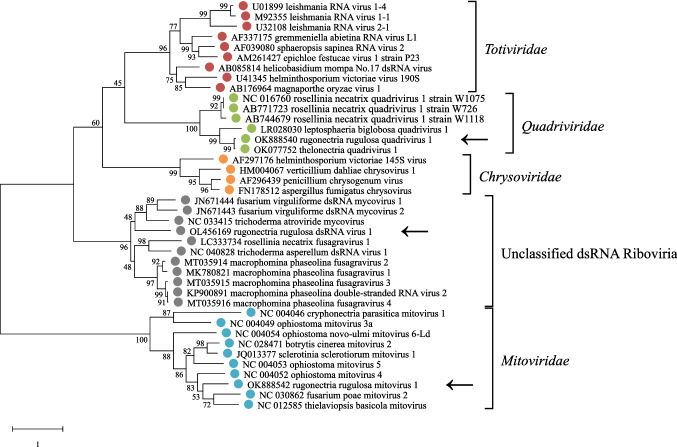
Phylogenetic tree, based on the alignment of conserved amino acids (aa) of RNA-dependent RNA polymerases (RdRp), referring to aa 679–1066 of the quadrivirus reference NC_016760. Alignment was performed with the MUSCLE algorithm [27]. The tree was constructed with the Maximum Likelihood method and 1000 bootstraps, using the program MEGA X [28]. The substitution model Le_Gascuel_2008 was used with discrete Gamma distribution (LG + G) [30]. Virus families are provided with colored dots and annotation of the family name. The scale bar is representing the substitutions per site. Numbers, next to the branches are indicating the percentage of trees, bootstrapped in the shown manner. Viruses are annotated with their GenBank Accession Number and virus name

For the taxonomic studies, the viruses with the highest BLAST scores to RrQV1, RrMV1, and RrV1 were selected. In addition, representatives of the families *Chrysoviridae* and *Totiviridae* were included in the calculations as further mycoviral entities. After RdRp translation and alignment, the most conserved region, represented by amino acids 679–1066 of NC_016760 was selected for each virus, as described by Chiba et al. [29]. This set of reduced-length sequences was used for the further alignment, based on which a phylogenetic tree was created in MEGA X [28]. Based on the tree, shown in Fig. 5 it can be stated that all three introduced viruses belong to their proposed family. RrQV1 clusters together with all other *Quadriviridae* and has the closest relation to OK077752 in 99% of 1000 bootstraps. The placement of RrQV1 in this cluster reinforces the assignment of this virus to the family. Moreover, the taxonomic proximity of *Chrysoviridae*, *Totiviridae*, and *Quadriviridae* has been shown previously and can be confirmed here [29, 43]. The mitovirus RrMV1 has the closest relation to NC_004052 and a subtree of NC_030862 and NC_012585. It is assigned to the family *Mitoviridae*, which form their own subtree with two clusters in 100% of the bootstraps. Since these + ssRNA viruses are much simpler in structure than the listed dsRNA viruses and use the genetic code of mold fungi, they were expected to form a distinct group and have a higher taxonomic distance from the other viruses [44, 45]. For this reason, no further outgrouper was necessary for the construction of the phylogenetic tree. According to that tree, RrV1 is assigned to the group of unclassified dsRNA *Riboviria*. In 96% of the bootstraps, it is listed together with JN671443, JN671444, and NC_033415 to a subtree of this group. The other subtree among the unclassified viruses summarizes mainly *Fusagravirus* species. RrV1 is therefore not included in the cluster, even if it could be considered as a possible family of its own in the future [46].

### Sequence identities and species demarcation

To determine the sequence identities of the new viruses in relation to the others in the phylogenetic tree, pairwise sequence alignments of the gRNA, RdRp-ORFs, and RdRp amino acid sequences were made. Results of analyses of the RdRp encoding Segment 3 of the quadrivirus RrQV1 with other members of the family led to a high similarity with thelonectria quadrivirus 1 (TQV1), with a protein-sequence identity of 94%. The lowest identity was found in the aa-sequence with RnQV1, isolates W1075 and W726 (30.1%). Table 3 gives an overview of all results, comparing segment 3 of the *Quadriviridae*. Since the identity of RrQV1 Segment 3 was comparatively high with TQV1, the remaining three segments were analyzed as well and summarized in Table 3. RnQV1 W726 was not included here, because of missing sequence data on segments 1, 2, and 4. As for segment 3, TQV1 has the highest similarities with RrQV1 in the other segments. The protein-sequence identity of those viruses varies from 69.9 to 82.8%. The lowest score was obtained with RnQV1 W1118, segment 1 (18.9%). For the determination of the species, there is no official demarcation value for the family of *Quadriviridae* [29]. The criteria in other virus families are very diverse and are therefore not suitable for transmission and application in this family. To date, only three different members of the family *Quadriviridae* are known. All of these viruses have been identified in different hosts. With RrQV1, for the first time, an infection within the genus *Rugonectria* could be detected. Therefore, we recommend RrQV1 as a member of a new species, tentatively named *Quadrivirus rugonectria* within the family *Quadriviridae*.

**Table 3:** Lengths and sequence identities (%) of rugonectria rugulosa quadrivirus 1 (RrQV1) dsRNA segments 1, 2, 3, and 4 with related *Quadriviridae*

Segment	Virus	Accession number	gRNA		Open reading frame		Protein sequence	
			Length (nt)	Identity (%)	Length (nt)	Identity (%)	Length (aa)	Identity (%)
**1**	**RrQV1**	**OK888538**	**4897**	**–**	**4776**	**–**	**1591**	**–**
1	RnQV1 W1075	NC_016757	4942	45.20	4809	44.10	1602	40.70
1	RnQV1 W1118	AB744677	4971	45.70	4809	45.70	1602	18.90
1	LbQV-1	LR028028	4728	45.80	4680	46.30	1559	23.00
1	TQV1	OK077750	4876	66.90	4776	67.00	1591	69.90
**2**	**RrQV1**	**OK888539**	**4312**	**–**	**4155**	**–**	**1384**	**–**
2	RnQV1 W1075	NC_016759	4352	44.90	4071	45.10	1356	20.50
2	RnQV1 W1118	AB744678	4307	45.70	4074	45.70	1357	21.30
2	LbQV-1	LR028029	4543	46.00	4152	46.30	1383	26.90
2	TQV1	OK077751	4312	73.30	4155	73.50	1384	82.80
3	**RrQV1**	**OK888540**	**4153**	**–**	**4056**	**–**	**1351**	–
3	RnQV1 W1075	NC_016760	4099	46.70	3933	47.50	1310	30.10
3	RnQV1 W726	AB771723	4104	47.30	3933	47.70	1310	30.10
3	RnQV1 W1118	AB744679	4093	48.20	3933	48.60	1310	30.30
3	LbQV-1	LR028030	4490	48.40	4104	50.60	1367	39.20
3	TQV1	OK077752	4158	80.70	4056	80.80	1351	94.00
**4**	**RrQV1**	**OK888541**	**3804**	**–**	**3363**	**–**	**1120**	**–**
4	RnQV1 W1075	NC_016758	3685	45.40	3186	44.80	1061	21.70
4	RnQV1 W1118	AB744680	3468	45.30	3180	46.00	1059	19.90
4	LbQV-1	LR028031	4048	45.40	3336	48.00	1111	27.60
4	TQV1	OK077753	3933	67.60	3363	71.90	1120	81.60

Bold values indicate the viruses identified in this study (RrQV1, RrMV1, RrV1)

Identities of the mitovirus RrMV1 with other species vary from 22% (aa-sequence, CpMV1) to 55.3% (ORF, BcMV-2). The highest similarity of the RrMV1 aa-sequence (38.4%) was found with CeMV (NC_012585). Table 4 sums up the gRNA-, ORF- and protein sequencesʹ lengths and identities of all analyzed *Mitoviridae* members. Following the change in taxa, mitoviruses are no longer assigned to the *Narnaviridae* as of 2019 but have their own family with the *Mitoviridae* [47, 48]. There is no species demarcation for the *Mitoviridae* yet. However, since RrMV1 is below 50% sequence identity in protein sequences compared to all other mitoviruses considered and this was the species demarcation value within the *Narnaviridae*, we propose it as member of a new species, tentatively named *Mitovirus rugonectria* within the family *Mitoviridae* [49].

**Table 4:** Lengths and sequence identities (%) of rugonectria rugulosa mitovirus 1 (RrMV1) gRNA, ORF coding for RdRp and RdRp protein sequence with related members in the genus *Mitovirus*

Virus	Accession number	gRNA		Open reading frame		Protein sequence	
		Length (nt)	Identity (%)	Length (nt)	Identity (%)	Length (aa)	Identity (%)
**RrMV1**	**OK888542**	**2410**	**–**	**2145**	**–**	**714**	**–**
CpMV1	NC_004046	2728	44.90	2103	47.30	700	22.00
OnuMV4	NC_004052	2599	53.90	2352	54.00	783	31.90
OnuMV5	NC_004053	2474	52.40	2190	54.00	729	33.30
OnuMV6Ld	NC_004054	2843	50.90	2088	52.40	695	28.10
OnuMV3a	NC_004049	2617	46.70	2157	47.20	718	23.20
SsMV1	JQ013377	2513	50.90	2076	51.20	691	36.60
CeMV	NC_012585	2896	49.90	2118	52.20	705	38.40
BcMV-2	NC_028471	2497	53.20	2133	55.30	710	38.30
FpMV1	NC_030862	2414	51.80	2295	51.60	764	37.70

Bold values indicate the viruses identified in this study (RrQV1, RrMV1, RrV1)

When comparing the RdRp aa-sequences of the unclassified RrV1 with other unclassified dsRNA *Riboviria* viruses, identities range from 29.3% (MpDSRV2) to 32.9% (FvV1, MpFV3). Similarities of gRNAs and ORFs are between 42.5% and 48.1%. In Table 5, all calculated sequence identities of RrV1 with other viruses are listed. Since all viruses similar in BLAST are unclassified, RrV1 should be assigned to the unclassified dsRNA *Riboviria* as well. For unclassified viruses, there are no guidelines for species demarcation. At less than 40%, the amino acid sequence identities of RdRp are also low compared to the other unclassified viruses. Therefore, RrV1 is proposed as a new virus. However, RrV1 cannot be assigned to a (new) species according to the ICTV guideline on naming viruses and virus species, since a new species must be assigned to a genus for binomial naming [50].

**Table 5:** Lengths and sequence identities (%) of rugonectria rugulosa dsRNA virus 1 (RrV1) gRNA, ORF coding for RdRp and RdRp protein sequence with related unclassified *Riboviria*

Virus	Accession number	gRNA		Open reading frame 2		Protein sequence	
		Length (nt)	Identity (%)	Length (nt)	Identity (%)	Length (aa)	Identity (%)
**RrV1**	**OL456169**	**8964**	**–**	**4086**	**–**	**1361**	**–**
TaMV1	NC_033415	8566	46.20	3645	45.80	1214	31.20
FvV1	JN671444.1	9402	46.70	3870	48.10	1289	32.90
MpFV2	MT035914.1	9024	45.70	3813	45.30	1270	32.80
MpDSRV2	KP900891.1	9188	45.40	3249	42.50	1082	29.30
MpFV3	MT035915.1	9328	44.70	3951	46.00	1316	32.90
RnFGV1	LC333734.1	9368	45.40	3936	47.90	1311	32.70
MpFV1	MK780821.1	9289	45.20	3813	46.70	1270	32.10
FvV2	JN671443.1	9327	46.70	3933	46.90	1310	32.70
TaRV1	NC_040828.1	9838	44.10	3987	46.30	1328	31.40
MpFV4	MT035916.1	8930	44.60	3627	45.90	1208	32.00

Bold values indicate the viruses identified in this study (RrQV1, RrMV1, RrV1)

## Conclusion

For the first time, three new viruses were identified using dsRNA extraction and Illumina sequencing from an endophytic *Rugonectria rugulosa* isolate associated with ARD. Full-length genomic RNAs were used to tease out the particular features of each virus. By phylogenetic analysis, rugonectria rugulosa quadrivirus 1, as member of the suggested species *Quadrivirus rugonectria* and rugonectria rugulosa mitovirus 1, as member of the proposed species *Mitovirus rugonectria* could be assigned to a taxonomic family. Together with rugonectria rugulosa dsRNA virus 1, all three viruses can be proposed as new viruses after analysis of sequence identities with related species. Future experiments need to clarify whether infections with these viruses have a hypo- or hypervirulent effect on the host. Although some mycoviral infections do not appear to have any effect on host infection behavior, there are reports of such virulence increasing and decreasing effects of some mycoviruses [9–15]. If any of these cases occur, the viruses could serve as a control target or biocontrol agent in the containment of ARD. Building on this, the effect of infection on the etiology of ARD needs to be elaborated. Thus, the presented results can contribute to the growing field of mycovirology and the diversity and spread of these viruses in plant root-associated fungi.

## Supplementary Information

Below is the link to the electronic supplementary material.

Supplementary file1 (PDF 11 kb)

Supplementary file2 (FASTA 45 kb)

Supplementary file3 (FASTA 19 kb)

Supplementary file4 (FASTA 41 kb)

Supplementary file5 (FASTA 38 kb)

Supplementary file6 (FASTA 37 kb)

Supplementary file7 (FASTA 36 kb)
