# Supplementary material for: Three new mycoviruses identified in the apple replant disease (ARD)-associated fungus Rugonectria rugulosa
Source: Virus Genes. 2022 Jul 16;58(5):423–35. doi: 10.1007/s11262-022-01924-6 (PMC9477930; doi:10.1007/s11262-022-01924-6)
Supplement: Supplementary file 1 — Supplementary file1 (PDF 11 kb) [file 11262_2022_1924_MOESM1_ESM.pdf]

Suppl. Table 1: Number of reads mapping the genomic RNAs of the mycoviruses identified in this study. A total of 5,281,812 reads were generated from sequencing of the library.

Virus	Genomic RNA	Length (nt)	Mapped reads	% of mapped reads
RrQV1	1	4897	236274	4.47
	2	4312	187644	3.55
	3	4153	177090	3.35
	4	3804	116304	2.20
RrMV1	1	2410	131223	2.48
RrV1	1	8964	477883	9.05
